# Supplementary material for: High-Throughput Determination of Multiclass Chemical Hazards in Poultry Muscles and Eggs Using UPLC–MS/MS
Source: Foods. 2025 May 8;14(10):1660. doi: 10.3390/foods14101660 (PMC12111322; doi:10.3390/foods14101660)
Supplement: Supplementary file 1 [file foods-14-01660-s001.zip › foods-3579908-supplementary.pdf]

Table S1 Mass spectrometry parameters, linear ranges and linear correlation coefficients of 280 targets and 44 internal standards

No.	Compounds	ESI	Retention time (min)	Precursor ion (*m*/*z*)	Product ions (*m*/*z*)	Collision energies/eV	Eggs		Chicken		LOD（μg/kg）	LOQ（μg/kg）	Internal standard
							Linear range /(μg/L)	R^2	Linear range /(μg/L)	R^2			
/	**Sulfonamides**												
1	Trimethoprim	+	6.89	291	261*/230	25/15	0.04~100	0.9989	0.04~100	0.9965	0.05	0.1	Sulfachlorpyridazine-$^{13}C_6$
2	Sulfaguanidine	+	3.41	215	156*/108	15/20	1~100	0.9974	1~100	0.9941	0.5	1	Sulfachlorpyridazine-$^{13}C_6$
3	Sulfapyridine	+	5.66	250	156*/184	16	0.04~100	0.9993	0.04~100	0.9990	0.2	0.5	Sulfadiazine-D_4
4	Sulfadiazine	+	4.63	251	156*/108	15/15	0.2~100	0.9972	0.1~100	0.9923	0.2	0.5	Sulfadiazine-D_4
5	Sulfamethoxazole	+	10.4	254	156*/108	14/14	0.2~100	0.9987	0.2~100	0.9978	0.5	1	Sulfamethoxazole-D_4
6	Sulfathiazole	+	5.45	256.1	156*/108	20/20	0.2~100	0.9968	0.2~100	0.9990	0.2	0.5	Sulfadiazine-D_4
7	Sulfamerazine	+	5.98	265.1	156*/172	15/15	0.04~100	0.9995	0.04~100	0.9995	0.2	0.5	Sulfachlorpyridazine-$^{13}C_6$
8	Sulfisoxazole	+	12.41	268	156*/113	14/16	0.2~100	0.9991	0.2~100	0.9980	0.2	0.5	Sulfamethoxazole-D_4
9	Sulfamoxole	+	12.39	268.1	156*/113.1	16/14	0.1~100	0.9993	0.1~100	0.9989	0.2	0.5	Sulfamethoxazole-D_4
10	Sulfamethizole	+	7.96	271	156*/108	14/14	0.1~100	0.9982	0.1~100	0.9990	0.5	1	Sulfachlorpyridazine-$^{13}C_6$

11	Sulfabenzamine	+	13.47	276.9	156*/108	15/15	0.04~100	0.9978	0.04~100	0.9977	0.2	0.5	Sulfamethoxazole-D_4
12	Sulfamethazine	+	7.42	279	186*/124	16/16	0.04~100	0.9996	0.04~100	0.9994	0.5	1	Sulfachlorpyridazine-$^{13}C_6$
13	Sulfamonomethoxine	+	9.34	281	156*/126	16/16	0.1~100	0.9976	0.1~100	0.9970	0.2	0.5	Sulfachlorpyridazine-$^{13}C_6$
14	Sulfachlorpyridazine	+	9.12	285.1	155.9*/108.1	12/12	0.2~100	0.9953	0.2~100	0.9932	0.2	0.5	Sulfachlorpyridazine-$^{13}C_6$
15	Sulfathinoxaline	+	16.01	301.1	156*/107.9	15/15	0.04~100	0.9907	0.1~100	0.9902	0.2	0.5	Sulfamethoxazole-D_4
16	Sulfadoxine	+	11.28	311	156*/108	15/15	0.04~100	0.9997	0.04~100	0.9990	0.2	0.5	Sulfamethoxazole-D_4
17	Sulfadimidine	+	7.16	279	124*	19	1~100	0.9995	1~100	0.9977	0.2	0.5	Sulfachlorpyridazine-$^{13}C_6$
18	Sulfacetamide	+	2.71	215	156*/92.2	10/20	1~100	0.9987	1~100	0.9961	0.5	1	Sulfadiazine-D_4
19	Sulfaphenazole	+	15.13	315.1	158.2*/92.2	28/30	0.2~100	0.9974	0.2~100	0.9987	0.05	0.1	Sulfamethoxazole-D_4
20	*N*-Acetylsulfamethoxazole	+	7.72	271	156*/108	22/35	0.2~100	0.9983	0.1~100	0.9978	0.5	1	Sulfachlorpyridazine-$^{13}C_6$
21	Sulfadimethoxine	+	15.54	311	108*	20	0.1~100	0.9932	0.1~100	0.9978	0.05	0.1	Sulfadimethoxine-$^{13}C_6$
22	Sulfameter	+	9.47	281	156*/126	16/16	0.1~100	0.9921	0.1~100	0.9937	0.2	0.5	Sulfamethoxazole-D_4
23	Sulfamethoxypridazine	+	7.75	281	156*/126	16/16	0.1~100	0.9900	0.1~100	0.9980	0.2	0.5	Sulfachlorpyridazine-$^{13}C_6$
24	Sulfisomidine	+	7.79	279	186	16	0.04~100	0.9933	0.04~100	0.9975	0.2	0.5	Sulfachlorpyridazine-$^{13}C_6$
/	**Quinolones**												
25	Pipemdilic acid	+	6.13	304.1	217*/189	21/32	0.4~100	0.9961	0.04~100	0.9959	0.5	1	Enrofloxacin-D_5

26	Sarafloxacin	+	12.2	386.1	298.9*/342.02	26/16	0.1~100	0.9975	0.1~100	0.9901	0.05	0.1	Sarafloxacin-D_8
27	Sparfloxacin	+	13.48	393.1	349.1*/291.9	18/22	0.04~100	0.9998	0.04~100	0.9998	0.2	0.5	Sarafloxacin-D_8
28	Nalidixic acid	+	17.81	233	215*/187	10/20	0.04~100	0.9995	0.04~100	0.9991	0.5	1	Sarafloxacin-D_8
29	Oxolinlic acid	+	18.90	262	244*/201.77	16/28	0.04~100	0.9991	0.04~100	0.9994	0.05	0.1	Sarafloxacin-D_8
30	Flumequine	+	18.08	262.2	261.8*/243.7	6/15	0.04~100	0.9991	0.04~100	0.9968	0.05	0.1	Sarafloxacin-D_8
31	Norfloxacin	+	7.78	320.1	233.2*/276.2	25/15	1~100	0.9998	1~100	0.9906	0.5	1	Enrofloxacin-D_5
32	Enoxacin	+	7.32	321.1	303.1*/234.1	19/22	0.04~100	0.9945	0.04~100	0.9953	0.8	2	Enrofloxacin-D_5
33	Pefloxacin	+	8.69	334.2	290.0*/232.9	16/22	0.04~100	0.9986	0.04~100	0.9977	0.8	2	Enrofloxacin-D_5
34	Lomefloxacin	+	9.34	352.2	308.0*/264.9	16/20	0.04~100	0.9995	0.1~100	0.9981	0.5	1	Enrofloxacin-D_5
35	Danolfloxacin	+	9.51	358.1	339.9*/95.9	22/20	0.04~100	0.9982	0.04~100	0.9980	0.05	0.1	Enrofloxacin-D_5
36	Ofloxacin	+	7.7	362.2	260.9*/318.1	26/16	0.04~100	0.9976	0.04~100	0.9901	0.5	1	Enrofloxacin-D_5
37	Enrofloxacin	+	9.87	360.1	316.1*/244.9	16/24	0.04~100	0.9987	0.04~100	0.9981	0.05	0.1	Enrofloxacin-D_5
38	Marbofloxacin	+	6.9	363	72*/320	18/14	0.04~100	0.9996	0.04~100	0.9985	0.5	1	Enrofloxacin-D_5
39	Fleroxacin	+	7.29	370.2	325.9*/268.9	16/26	0.04~100	0.9993	0.04~100	0.9989	0.8	2	Enrofloxacin-D_5
40	Gatifloxacin	+	12.41	376.2	332*/	16/30	0.04~100	0.9992	0.04~100	0.9987	0.2	0.5	Sarafloxacin-D_8

41	Pazufloxacin	+	8.71	319.1	302*/284	10/18	1~100	0.9981	1~100	0.9915	4	10	Enrofloxacin-D_5
42	Difloxacin	+	12.23	400	356*/382	17/18	0.04~100	0.9995	1~100	0.9997	0.05	0.1	Sarafloxacin-D_8
43	Ciprofloxacin	+	8.29	332.1	288*/244.9	16/22	1~100	0.9954	1~100	0.9916	4	10	Enrofloxacin-D_5
/	**Tetracycline**												
44	Minocycline	+	6.9	458.2	441.2*/283.2	18/40	2~100	0.9951	2~100	0.9987	8	20	/
/	**Anti-coccidials**												
45	Monensin	+	24.94	693.3	675.1*/461.1	35/40	0.4~100	0.9921	0.2~100	0.9942	0.8/0.5#	2/1#	/
46	Salinomycin	+	25.09	773.6	431.2*/531.1	40/35	0.4~100	0.9978	0.4~100	0.9912	0.5	1	/
47	Narasin	+	25.23	787.6	431.3/279.1	40/45	2~100	0.9958	1~100	0.9965	1/0.5#	2/1#	/
48	Maduramicin	+	25.13	939.8	473.5*/451.4	72/75	1~100	0.9977	2~100	0.9900	2/5#	5/10#	/
49	Diaveridine	+	6.21	261.2	123.2*/245.2	20/30	0.1~100	0.9987	0.1~100	0.9987	0.5	1	Dimetridazole-D_3
50	Guanabenz	+	21.73	334.2	154.8*/137.8	18/20	0.04~100	0.9991	0.04~100	0.9978	0.5	1	/
51	Doramectin	+	24.83	899.5	113.2*/219.2	30/15	4~100	0.9927	4~100	0.9904	8	20	/
52	Ethopabate	+	16.25	238.1	205.8*/163.7	10/20	0.04~100	0.9993	0.04~100	0.9997	0.2	0.5	/
53	Robenidine	+	21.74	334.1	138.2*/155.2	25/18	0.1~100	0.9900	0.1~100	0.9910	0.5	1	/
54	Rafoxanide	+	24.78	625.8	127*/373	30/30	0.1~100	0.9931	0.1~100	0.9921	0.5	1	/

55	Clopidol	+	5.34	192	87.2*/101.2	30/25	1~100	0.9973	1~100	0.9930	4	10	Dimetridazole-D_3
56	Levamisol	+	5.52	205.1	178*/91	20/30	0.04~100	0.9998	0.04~100	0.9998	0.2	0.5	Dimetridazole-D_3
57	Triclabendazole	+	23.37	359	274*/309	36/30	0.04~100	0.9998	0.04~100	0.9976	0.2	0.5	/
58	Decoquinate	+	24.4	418.5	232.2*/390.4	35/20	0.04~100	0.9995	0.04~100	0.9954	0.2	0.5	Tilmicosin-D_3
59	Febantel	+	22.81	447.1	383.1*/415.2	12/18	0.04~100	0.9955	0.04~100	0.9945	0.2	0.5	Tilmicosin-D_3
60	Nicarbazin	-	4.34	301	107*/137	35/11	0.04~100	0.9984	0.04~100	0,9984	0.5	1	Nicarbazin-D_8
61	Diclazuril	-	4.57	405	299*/334	12/16	0.04~100	0.9901	0.04~100	0.9986	0.2	0.5	Toltrazuril-D_3
62	Toltrazuril sulfoxide	-	5.44	440.2	371.2*/383.2	25/15	0.1~100	0.9983	0.1~100	0.9945	0.5	1	Toltrazuril-D_3
63	Fipronil-sulfide	-	6.37	419. 3	383*	10	0.04~100	0.9906	0.04~100	0.9900	0.5	1	Fipronil-$^{13}C_2$, $^{15}N_2$
64	Toltrazuril	-	5.42	424	424*	10	0.2~20	0.9957	0.2~100	0.9904	0.2	0.5	Toltrazuril-D_3
65	Ponazuril	-	4.40	456.2	399*	12	2~40	0.9901	2~100	0.9978	4	10	Toltrazuril-D_3
/	**Macrolides**												
66	Timicosin	+	18.06	869.6	174.2*/696.5	45/40	2~100	0.9932	2~100	0.9900	5	10	Tilmicosin-D_3
67	Lincomycin	+	6.35	407.2	126.2*/359.3	28/18	0.04~100	0.9985	0.04~100	0.9982	0.8	2	Lincomycin-D_3
68	Virginiamycin m1	+	21.39	526.2	355.1*/508.1	17/14	0.04~100	0.9990	0.04~100	0.9921	0.5	1	Eryhronycin-^{13}C-D_3
69	Oleandonycin	+	18.47	688.4	158.2*/544.4	28/18	0.04~100	0.9991	0.04~100	0.9965	0.2	0.5	Eryhronycin-^{13}C-D_3
70	Eryhronycin	+	19.43	734.5	158.2*/576.5	30/18	0.04~100	0.9972	0.1~100	0.9932	0.2/0.5$^{\#}$	1	Eryhronycin-^{13}C-D_3

71	Azithromycin	+	16.27	749.5	158.2*/591.5	38/30	0.04~100	0.9982	0.04~100	0.9914	0.2	0.5	Azithromycin-D_3
72	9-Deoxo-9a-aza-9a-home erythomycin A	+	19.43	735.5	158.2*/577.6	35/25	1~100	0.9984	2~100	0.9912	5/10#	20	Eryhronycin-^{13}C-D_3
73	Kitasamycin	+	21.11	786.5	109.1*/174.2	40/30	1~100	0.9951	1~100	0.9913	0.2	0.5	Eryhronycin-^{13}C-D_3
74	Telithromycin	+	16.86	812.5	655.58*/158.2	32/40	0.2~100	0.9932	0.2~100	0.9982	0.2	0.5	Azithromycin-D_3
75	Midecamycin	+	21.06	814.5	109*/174	45/35	0.04~100	0.9994	0.04~100	0.9935	0.5	1	Eryhronycin-^{13}C-D_3
76	Rifampicin	+	22.4	823.4	791.6*/399.2	18/25	0.04~100	0.9916	0.1~100	0.9921	0.5	1	Eryhronycin-^{13}C-D_3
77	Viginamycin S1	+	22.36	824.3	205*/190	40/35	0.2~100	0.9950	0.2~100	0.9901	0.5	1	Eryhronycin-^{13}C-D_3
78	Roxithromycin	+	21.47	837.5	158.2*/679.7	35/20	0.04~100	0.9987	0.04~100	0.9921	0.5	1	Eryhronycin-^{13}C-D_3
79	Spiramycin	+	16.12	843.5	174.2*/101.2	35/40	1~100	0.9943	1~100	0.9900	0.5	1	Azithromycin-D_3
80	Tylosin	+	20.02	916.5	174.2*/101.2	35/45	1~100	0.9912	1~100	0.9903	4	10	Eryhronycin-^{13}C-D_3
81	Acetylkitasamycin	+	22.26	856.5	109.1*/174.2	38/30	0.04~100	0.9981	0.04~100	0.9911	0.2	0.5	Eryhronycin-^{13}C-D_3
82	Desmycosin	+	18.92	772.4	174.1*/98.2	28/40	0.4~100	0.9978	0.4~100	0.9978	0.5	1	Eryhronycin-^{13}C-D_3
/	**Nitroimidazoles**												
83	Dimetridazole	+	4.41	142.1	96*/81	16/20	0.2~100	0.9967	0.2~100	0.9984	0.2	0.5	Dimetridazole-D_3

84	Metronidazole	+	3.94	172	128*/82	14/20	0.04~100	0.9968	0.04~100	0.9988	0.05	0.1	Metronidazole$^{15}N^{13}C$
85	Ronidazole	+	4.49	201	140*/55	10/10	0.04~100	0.9978	0.04~100	0.9913	0.05	0.1	Ronidazole-D_3
86	Tinidazole	+	6.12	248	121*/128	12/18	0.2~100	0.9931	0.2~100	0.9983	0.2	0.5	Dimetridazole-D_3
87	Onidazole	+	7.77	220	128.2*/82.2	15/25	0.4~100	0.9988	0.2~100	0.9962	1/0.5#	2/1#	Dimetridazole-D_3
88	Aminitrozole	+	10.73	188	146*/100.2	10/20	0.04~100	0.9986	0.04~100	0.9901	0.5	1	Ronidazole-D_3
89	4-Nitroimidazole	+	1.48	114	68*/84	10/14	1~100	0.9972	1~100	0.9945	4	10	Dimetridazole-D_3
90	2-Methyl-5-nitroimidazole	+	2.26	128	82*/42	15/18	0.4~100	0.9936	0.4~100	0.9938	0.5	1	Metronidazole-hydroxy-D_4
91	5-Chloro-1-methyl-4-nitroimidazole	+	5.64	162	116*/81	15/20	0.2~100	0.9995	0.2~100	0.9990	0.5	1	Metronidazole-hydroxy-D_4
92	5-Nitrobenzimidazole	+	5.63	164	118*/91	15/18	0.04~100	0.9983	0.04~100	0.9992	0.5	1	Metronidazole-hydroxy-D_4
93	Ipronidazole	+	10.62	170	124*/109	14/20	0.2~100	0.9968	0.2~100	0.9986	4	10	Dimetridazole-D_3
94	Metronidazole-hydroxy	+	2.58	188	126*/144	10/14	0.4~100	0.9964	0.4~100	0.9988	4	10	Metronidazole-hydroxy-D_4
95	Dimetridazole-2-hydroxy	+	3.41	158	140*/55	10/16	4~100	0.9912	4~100	0.9933	8	20	Metronidazole-hydroxy-D_4
/	**Benzimidazoles**												
96	Cambendazole	+	15.26	303	217.2*/243.2	28/30	0.2~100	0.9994	0.1~100	0.9978	0.3/0.2#	1/0.5#	/

97	Oxibendazole	+	15.57	250.1	218.1*/148.2	18/35	0.04~100	0.9994	0.04~100	0.9997	0.2	0.5	/
98	Flubendazole	+	19.35	314.1	282.2*/123	18/35	0.1~100	0.9984	0.1~100	0.9960	0.5	1	/
99	Albendazole	+	18.9	266	234*/191	20/32	0.04~100	0.9972	0.04~100	0.9986	0.2	0.5	/
100	Albendazole sulfoxide	+	11.65	282.1	240*/159	12/32	1~100	0.9991	1~100	0.9991	4	10	Ronidazole-D_3
101	Fenbendazole sulfone	+	17.7	332	300.2*/159	22/35	0.2~100	0.9992	0.2~100	0.9992	0.5	1	/
102	Oxfendazole	+	15.85	316	191.2*/284	20/18	0.4~100	0.9984	0.4~100	0.9992	0.5	1	Dimetridazole-D_3
103	Fenbendazole	+	21.38	300	268.2*/159.2	20/35	0.04~100	0.9992	0.04~100	0.9964	0.5	1	/
104	5-Hydroxymebendazole	+	14.2	298.1	160.2*/266.2	35/35	0.4~100	0.9989	0.4~100	0.9987	0.5	1	/
105	2-Aminoflubendazole	+	14.91	256.1	123.2*/95	25/38	0.04~100	0.9994	0.1~100	0.9992	0.5	1	/
106	5-Hydroxythiabendazole	+	5.6	218	191.1*/147.2	25/30	0.2~100	0.9966	0.2~100	0.9992	0.2	0.5	Dimetridazole-D_3
107	Mebendazole-amine	+	13.97	238.1	105.2*/133.2	25/35	0.4~100	0.9900	0.4~100	0.9990	2	5	/
108	Albendazole-2-aminosulfone	+	6.00	240	133.1*/198	30/25	0.4~100	0.9976	0.4~100	0.9982	4	10	Dimetridazole-D_3
109	Thiabendazole	+	6.27	202	175.2*/131.2	25/30	0.04~100	0.9989	0.04~100	0.9992	0.2	0.5	Dimetridazole-D_3
/	**Chloramphenicols**												
110	Thiamphenicol	-	2.23	354.1	185*/290.	12/18	0.04~100	0.9956	0.04~100	0.9901	0.5	1	Thiamphenicol-D_3

111	Chloramphenicol	-	2.86	321	152*/257	11/17	0.04~100	0.9925	0.04~100	0.9998	0.05	0.1	Chloramphenicol-D_5
112	Florfenicol	-	2.73	356	185*/336	10/18	0.04~100	0.9997	0.04~100	0.9991	0.05	0.1	Florfenicol-D_3
/	**Antifungals**												
113	Clotrimazole	+	21.05	277.2	242*/166	18/18	0.1~100	0.9956	0.1~100	0.9968	0.5	1	/
114	Fluconazole	+	11.49	307.1	220*/238	18/16	0.4~100	0.9977	0.4~100	0.9934	2	5	/
115	Griseofulvin	+	20.8	353.1	165*/215	18/18	0.2~100	0.9987	0.04~100	0.9976	1/0.5#	2/1#	/
116	Econazol	+	22.15	381	125*/255	24/20	0.04~100	0.9954	0.04~100	0.9951	0.5	1	/
117	Ketoconazole	+	20.39	531.2	82*/498.1	40/30	0.2~100	0.9987	0.4~100	0.9979	0.4/0.6#	1/2#	/
118	Bifonazole	+	21.29	311.2	243*/165	12/30	0.04~100	0.9954	0.04~100	0.9943	0.5	1	/
/	**Beta-blockers**												
119	Isoxsuprine	+	14.4	302.2	284.2*/150	15/20	0.04~100	0.9988	0.1~100	0.9996	0.2/0.5#	0.5/1#	Oxprenolol-D_7
120	Pindolol	+	6.43	249.1	115.8*/171.8	14/14	0.04~100	0.9992	0.04~100	0.9998	0.5	1	Pindolol-D_7
121	Alprenolol	+	17.36	250.1	115.8*/71.8	15/17	0.04~100	0.9934	0.04~100	0.9997	0.5	1	Propranolol-D_7
122	Propranolol	+	17.08	260.2	115.8*/182.7	16/15	0.04~100	0.9988	0.04~100	0.9996	0.5	1	Propranolol-D_7
123	Oxprenolol	+	15.05	266.2	72.2*/116.2	20/15	0.04~100	0.9988	0.04~100	0.9998	0.2	0.5	Oxprenolol-D_7
124	Metoprolol	+	12.11	268.1	115.8*/190.7	14/14	0.4~100	0.9902	0.4~100	0.9951	0.5	1	Oxprenolol-D_7

125	Levobunolol	+	13.56	292.1	235.9*/200.9	19/19	0.04~100	0.9998	0.04~100	0.9994	0.2	0.5	Oxprenolol-D_7
126	Carazolol	+	15.08	299.2	115.8*/221.9	16/17	0.04~100	0.9989	0.04~100	0.9995	0.2	0.5	Oxprenolol-D_7
127	Betaxolol	+	17.73	308.1	115.8*/207.2	20/17	0.2~100	0.9978	0.04~100	0.9947	1/0.5#	2/1#	Propranolol-D_7
128	Timolol	+	11.43	317	261.1*/73.5	13/19	0.1~100	0.9997	0.1~100	0.9983	0.5	1	Acebutolol-D_5
129	Acebutolol	+	12.14	337.2	115.8*/319.1	18/14	0.04~100	0.9999	0.04~100	0.9998	0.5	1	Acebutolol-D_5
130	Atenolol	+	4.68	267.1	189.7*/115.8	16/16	1~100	0.9946	1~100	0.9985	0.5	1	Atenolol-D_7
131	Penbutolol	+	14.08	292.2	236*/73.7	13/18	0.04~100	0.9998	0.04~100	0.9997	0.2	0.5	Acebutolol-D_5
132	Nadolol	+	7.24	310	254*/235.9	20/25	0.2~100	0.9995	0.2~100	0.9996	0.5	1	Acebutolol-D_5
133	Nebivolol	+	20.5	406	150.8*/122.8	30/35	0.04~100	0.9992	0.04~100	0.9932	0.2	0.5	Propranolol-D_7
134	Carvedilol	+	19.02	407	99.8*/223.7	26/19	0.04~100	0.9981	0.04~100	0.9981	0.2	0.5	Chlorpromazine-D_6
/	**Sedatives**												
135	Azaperone	+	10.07	328.1	164.8*/120.8	18/17	0.04~100	0.9998	0.04~100	0.9994	0.5	1	Acebutolol-D_5
136	Azaperol	+	7.76	330.2	121.1*/149.2	35/40	0.04~100	0.9985	0.04~100	0.9952	0.5	1	/
137	Fupaiding	+	16.76	380.1	164.8*/193.7	24/13	0.04~100	0.9996	0.04~100	0.9985	0.2	0.5	Chlorpromazine-D_6
138	Estazolam	+	20.07	294.9	266.9*/191.6	25/20	0.04~100	0.9993	0.04~100	0.9996	0.2	0.5	Chlorpromazine-D_6
139	Chlorpromazine	+	20.94	319	85.8*/245.9	15/19	0.04~100	0.9974	0.04~100	0.9987	0.05	0.1	Chlorpromazine-D_6

140	Acepromazine	+	19.12	327.1	85.8*/253.8	17/21	0.1~100	0.9957	0.1~100	0.9984	0.5	1	Chlorpromazine-D_6
141	Benzocaine	+	15.15	166.1	138*/94	15/20	0.1~100	0.9938	0.1~100	0.9972	0.5	1	Chlorpromazine-D_6
142	Xylazine	+	10.05	221.1	89.7*/163.7	19/19	0.04~100	0.9996	0.04~100	0.9998	0.5	1	Chlorpromazine-D_6
143	Nitrazepam	+	19.49	282.1	235.9*/207.3	20/28	0.04~100	0.9976	0.1~100	0.9994	0.5	1	Diazepam-D_5
144	Diazepam	+	21.92	284.9	192.7*/153.6	25/22	0.2~100	0.9984	0.2~100	0.9946	0.05	0.1	Diazepam-D_5
145	Demoxepam	+	20.2	286.9	240.9*/269	20/15	0.4~100	0.9975	0.4~100	0.9903	0.5	1	Diazepam-D_5
146	Detomidine	+	13.39	187.1	81*/54	22/32	0.04~100	0.9995	0.04~100	0.9994	0.2	0.5	Diazepam-D_5
147	Diphenhydramine	+	17.69	256.2	167*/152	12/35	1~100	0.9963	0.1~100	0.9986	5/0.5#	10/1#	Diazepam-D_5
148	Bisacodyl	+	20.73	362.1	184*/226	28/18	0.04~100	0.9995	0.04~100	0.9990	0.2	0.5	Chlorpromazine-D_6
149	Clozapine	+	16.64	327.2	270.1*/192.1	20/38	0.04~100	0.9995	0.04~100	0.9958	0.2	0.5	Diazepam-D_5
150	Lidocaine	+	7.84	253.2	86*/58	18/32	0.1~100	0.9998	0.04~100	0.9972	1/0.5#	2/1#	Sulfachlorpyridazine-$^{13}C_6$
151	Chlordiazepoxide	+	6.32	300.1	283.1*/227.1	12/25	0.04~100	0.9921	0.04~100	0.9971	0.5	1	Sulfachlorpyridazine-$^{13}C_6$
152	Alprazolam	+	20.95	309.1	281.1/205.1	25/12	0.04~100	0.9994	0.04~100	0.9990	0.5	1	Chlorpromazine-D_6
153	Methadone	+	22.00	310	265*/105	15/25	0.2~100	0.9937	0.2~100	0.9900	0.5	1	Chlorpromazine-D_6
154	Triazolam	+	21.04	343	239*/308	45/30	0.04~100	0.9987	0.04~100	0.9986	0.5	1	Chlorpromazine-D_6
155	Benzthiazide	+	19.33	432	91*/65	20/70	1~100	0.9987	0.4~100	0.9967	5/2#	10/5#	Chlorpromazine-D_6

156	Fluphenazine	+	21.84	438	170.9/142.9	21/25	0.04~100	0.998	0.04~100	0.9976	0.05	0.1	Chlorpromazine-D_6
157	Paroxetine	+	7.84	330.2	192.1*/70.2	28/18	0.2~100	0.9990	0.2~100	0.9930	0.8	2	Sulfachlorpyridazine-$^{13}C_6$
158	Clonazepam	+	19.96	316.1	270*/86.2	20/15	0.04~100	0.9991	0.04~100	0.9965	0.05	0.1	diazepam-D_5
159	Lorazepam	+	20.62	321.2	275.1*/86.2	20/18	0.04~100	0.9905	0.04~100	0.9905	0.05	0.1	diazepam-D_5
/	**Hormones**												
160	Cortisone	+	18.53	361.2	163.2*/105.2	25/35	1~100	0.9942	1~100	0.9952	4	10	17β-boldenone-D_3
161	Prednisolone	+	18.68	361.2	307.4*/325.4	10/8	1~100	0.9951	1~100	0.9988	0.2	0.5	17β-boldenone-D_3
162	Hydrocortisone	+	18.73	363.2	121.1*/105.1	22/44	1~100	0.9952	1~100	0.9905	0.2	0.5	17β-boldenone-D_3
163	Betamethasone	+	20.17	393.2	373.2*/355.1	8/12	1~100	0.9969	1~100	0.9907	4	10	17β-boldenone-D_3
164	17α-Hydroxyprogesterone	+	21.89	331.2	97.2*/109	20/24	1~100	0.9900	1~100	0.9903	0.5	1	17β-boldenone-D_3
165	21α-Hydroxyprogesterone	+	22.26	331.2	97.2*/109	20/24	1~100	0.9912	1~100	0.9921	0.5	1	17β-boldenone-D_3
166	Trenbolone	+	20.36	271	199*/165.3	22/44	0.1~100	0.9921	0.1~100	0.9902	0.2	0.5	Testosterone-D_2
167	Levonorgestrel	+	22.4	313.1	108.9*/254.4	30/30	2~100	0.9900	2~100	0.9931	4	10	Levonorgestrel-D_6
168	Progesterone	+	23.16	315.2	97*/109	20/20	2~100	0.9912	2~100	0.9953	4	10	Progesterone-D_9
169	Megestrol acetate	+	23.07	385.1	267.1*/325.1	16/16	0.2~100	0.9988	0.2~100	0.9947	0.5	1	Megestrol-D_3
170	Androstendione	+	21.84	287.5	97.2*/109.2	20/24	2~100	0.9912	0.5~100	0.9947	2/0.5$^{\#}$	8/2$^{\#}$	Progesterone-D_9

171	Beclomethasone	+	20.55	409.5	373.4*/121.2	8/42	2~100	0.9901	2~100	0.9982	8	20	17β-boldenone-D_3
172	Triamcinolone acetonide	+	20.62	435.2	415.2*/397.2	10/12	0.4~100	0.9989	0.4~100	0.9972	0.8	2	17β-boldenone-D_3
173	Triamcinolone	+	14.15	395.2	357.2*/147.2	12/30	4~100	0.9928	4~100	0.9953	4	10	17β-boldenone-D_3
174	Methylprednisone	+	24.37	419.7	373.3*/343.60	12/19	1~100	0.9981	0.4~100	0.9947	4	10	Methyltestosterone-D_3
175	Norandrostenedione	+	21.22	273.2	108.9*/197.3	25/18	0.2~100	0.9931	0.2~100	0.9976	4	10	Progesterone-D_9
176	Dananzol	+	23.22	338.2	148*/120	25/30	1~100	0.9902	0.4~100	0.9967	0.5	1	Methyltestosterone-D_3
177	Methylandrostendiol	+	20.19	287.4	269.1*/159.1	11/21	2~100	0.9931	2~100	0.9963	8	20	Progesterone-D_9
178	Corticosterone	+	20.47	347.2	329.5*/121	15/20	2~100	0.9931	2~100	0.9987	4	10	17β-boldenone-D_3
179	Pregnenolone	+	22.18	317.2	299.2*/281.1	8/12	0.04~100	0.9912	0.04~100	0.9936	0.5	1	Methyltestosterone-D_3
180	Androstenediol	+	16.04	291.2	273.2*/231.2	10/18	0.4~100	0.9913	0.4~100	0.9900	4	10	Progesterone-D_9
181	Nandrolone phenylpropionate	+	24.42	407	105*/257	28/15	1~100	0.9912	1~100	0.9901	0.5	1	Methyltestosterone-D_3
182	Testosterone propionate	+	24.07	345	97*/109	20/22	1~100	0.9931	1~100	0.9931	2	5	Testosterone-D_2
183	Nandrolone propionate	+	23.86	331	257*/275	16/16	1~100	0.9931	1~100	0.9900	2	5	Methyltestosterone-D_3
184	Stanozolol	+	22.26	329	81*/91	42/40	0.2~100	0.9954	0.2~100	0.9986	2	5	Methyltestosterone-D_3
185	Methyltestosterone	+	22.2	303	109*/97	27/25	0.04~100	0.9951	0.04~100	0.9992	1	3	Methyltestosterone-D_3
186	Testosterone	+	21.76	289	97*/109	10/22	0.4~100	0.9931	0.4~100	0.9960	0.5	1	Testosterone-D_2

187	Boldenone	+	20.92	287	121*/135	22/15	1~100	0.9978	1~100	0.9967	0.5	1	17β-boldenone-D_3
188	Nandrolone	+	21.1	275.1	109*/257	28/15	0.2~100	0.9967	0.2~100	0.9900	2	5	Methyltestosterone-D_3
189	Medroxyprogesterone	+	23.19	387.1	327.1*/285.1	16/16	0.1~100	0.9974	0.04~100	0.9906	0.5/0.2#	1/0.5#	Methyltestosterone-D_3
190	Chlormadinone acetate	+	23.12	405.1	309.1*/345.1	12/16	1~100	0.9965	1~100	0.9965	0.5	1	Progesterone-D_9
191	Testosterone decanoate	+	25.64	443.5	109.2*/271.2	32/18	2~100	0.9931	2~100	0.9965	4	10	Testosterone-D_2
192	Testosterone undecanoate	+	25.28	457.4	97.1*/109.2	24/25	0.2~100	0.9985	0.2~100	0.9989	0.5	1	Testosterone-D_2
193	Testosterone phyenylpropionate	+	24.56	421.5	105.2*/97.2	28/25	0.1~100	0.9915	0.1~100	0.9912	0.5	1	Testosterone-D_2
194	Clobetasol propionate	+	23	467.2	355.2*/159.2	12/35	0.04~100	0.9931	0.04~100	0.9974	0.5	1	Testosterone-D_2
195	Fludrocortisone acetate	+	18.64	381	239.3*/91.2	25/58	4~100	0.9934	4~100	0.9967	4	10	Testosterone-D_2
196	Dexamethasone	+	20.15	393.2	355.2*/147.2	10/25	1~100	0.9994	1~100	0.9975	0.8	2	Testosterone-D_2
197	Hydroxyprogesterone	+	22.12	331.1	108.9*	20	2~100	0.9928	1~100	0.9966	0.5	1	Progesterone-D_9
198	Diethylstilbestrol	-	4.12	267.1	237*/251	28/25	0.04~100	0.9967	0.04~100	0.9960	0.2	0.5	Dienoestrol-D8
199	Estrone	-	3.97	269	145*/159	41/37	0.04~100	0.9908	0.04~100	0.9953	0.5	1	Estrone-2,3,4-$^{13}C_3$
200	Estradiol	-	3.60	271	145*/183	43/43	0.04~100	0.9956	0.04~100	0.9963	0.5	1	Estradiol-$^{13}C_2$
/	**Agonists**												

201	Clonidine	+	5.28	230	44.1*/167	22/30	0.04~100	0.9912	0.04~100	0.9993	0.2	0.5	Cimaterol-D_7
202	Phenylethanolamine A	+	18.1	345.2	327.2*/150.2	12/20	0.04~100	0.9983	0.04~100	0.9991	0.2	0.5	Clenbuterol-D_9
203	Cimaterol	+	4.11	220.1	160.1*/143	15/24	0.04~100	0.9912	0.04~100	0.9926	0.2	0.5	Cimaterol-D_7
204	Terbutaline	+	4.23	226.1	152*/125	16/26	0.2~100	0.9933	0.2~100	0.9925	0.5	1	Cimaterol-D_7
205	Salbutamol	+	3.86	240.2	148.1*/222.1	12/20	1~100	0.9900	1~100	0.9900	0.05	0.1	Salbutamol-D_3
206	Procaterol	+	5.36	291.2	273.2*/231.1	12/18	0.4~100	0.9991	0.4~100	0.9992	0.2	0.5	Cimaterol-D_7
207	Cimbuterol	+	5.39	234.2	160.1*/217	10/15	0.04~100	0.9900	0.04~100	0.9952	0.05	0.1	Cimaterol-D_7
208	Fenoterol	+	5.65	304.2	135.1*/107	20/18	0.1~100	0.9921	0.1~100	0.9991	0.05	0.1	Cimaterol-D_7
209	Clenproperol	+	7.72	263.1	245*/203	14/20	0.2~100	0.9947	0.2~100	0.9969	0.2	0.5	Clenbuterol-D_9
210	Clorprenaline	+	8.4	214	154*/118	18/22	0.04~100	0.9993	0.04~100	0.9900	0.05	0.1	Clorprenaline-D_7
211	Ractopamine	+	8.91	302.2	164.1*/284.2	12/15	0.1~100	0.9998	0.1~100	0.9960	0.05	0.1	Ractopamine-D_5
212	Clenbuterol	+	10.91	277.1	203*/132	15/15	0.2~100	0.9941	0.2~100	0.9910	0.05	0.1	Clenbuterol-D_9
213	Tulobuterol	+	11.94	228.1	154*/172	12/15	0.04~100	0.9998	0.04~100	0.9907	0.05	0.1	Clenbuterol-D_9
214	Formoterol	+	12.41	345.1	149*/327.1	14/18	0.04~100	0.9998	0.04~100	0.9998	0.05	0.1	Clenbuterol-D_9
215	Brombuterol	+	13.57	365.1	290.0*/217.1	20/25	0.04~100	0.9995	0.04~100	0.9991	0.05	0.1	Clenbuterol-D_9
216	Mabuterol	+	14.3	311.1	237.1*/217.1	15/25	0.04~100	0.9997	0.04~100	0.9997	0.05	0.1	Clenbuterol-D_9

217	Bambuterol	+	15.36	368.1	294.3*/312.3	10/16	0.04~100	0.9991	0.04~100	0.9997	0.05	0.1	Clenbuterol-D_9
218	Clenhexerol	+	18.42	304.9	287.2*/188.3	10/20	0.2~100	0.9901	0.4~100	0.9993	0.2/0.5#	0.5/1#	Clenbuterol-D_9
219	Salmeterol	+	21.19	416.2	398.3*/380.3	12/16	0.1~100	0.9913	0.1~100	0.9900	0.05	0.1	Salmeterol-D_3
220	Clenisopenterol	+	16.13	291	188*/216.9	22/16	0.1~100	0.9971	0.1~100	0.9988	0.2	0.5	Clenbuterol-D_9
221	Clencyclohenxerol	+	15.66	291.1	273*/188	10/20	0.4~100	0.9948	0.4~100	0.9965	0.2	0.5	Clenbuterol-D_9
222	Cyprohepatadine	+	19.68	288.2	96*/191	25/28	0.04~100	0.9996	0.04~100	0.9997	0.5	1	Clenbuterol-D_9
223	Clenpenterol	+	15.07	291.1	203*/273	10/15	0.04~100	0.9992	0.04~100	0.9997	0.2	0.5	Clenbuterol-D_9
/	**Antiinflammatory drugs**												
224	Flurbiprofen	+	23.5	245.1	115*/87	12/20	0.04~100	0.9992	0.04~100	0.9986	0.2	0.5	/
225	Fenbufen	+	21.89	255.1	237*/181	19/25	0.1~100	0.9977	0.1~100	0.9952	0.5	1	/
226	Indoprofen	+	20.57	282.1	236.3*/218.3	20/30	0.2~100	0.9964	0.2~100	0.9991	0.2	0.5	/
227	Tolmetin	+	21.19	258.1	119.2*/91.2	18/35	0.04~100	0.9965	0.1~100	0.9978	0.5	1	/
228	Mefenamic acid	+	23.44	242.1	224.2*/180.2	15/38	0.2~100	0.9963	0.2~100	0.9909	0.5	1	/
229	Flufenamic acid	+	12.43	282.1	236.3*/218.3	22/28	0.4~100	0.9908	0.2~100	0.9976	0.2	0.5	/
230	Indometacin	+	23.07	358.1	139.1*/174.2	12/18	1~100	0.9964	1~100	0.9913	2	5	/
231	Sulindac	+	21.07	357.1	233.2*/248.3	48/32	0.2~100	0.9985	0.2~100	0.9982	0.8	2	/

232	Piroxicam	+	18.61	332.1	95.2*/121.2	18/20	0.04~100	0.9964	0.04~100	0.9915	0.5	1	/
233	Meloxicam	+	21.29	352	115.2*/141.1	18/20	0.1~100	0.9956	0.1~100	0.9982	0.5	1	/
234	Etodolac	+	22.73	288.2	172*/143	10/40	1~100	0.9936	0.4~100	0.9987	1/0.5#	2/1#	/
235	Diclofenac	+	22.86	297	216*/251	12/18	2~100	0.9929	2~100	0.9939	4	10	/
/	**Hypoglycemic drugs**												
236	Glibenclamide	+	22.76	494.2	369*/304.2	15/28	0.2~100	0.9980	0.2~100	0.9927	0.2	0.5	/
237	Gliclazide	+	21.7	324.1	127*/153	18/20	0.04~100	0.9905	0.04~100	0.9942	0.5	1	/
238	Glimepiride	+	23.03	491.2	126*/352	12/25	0.4~100	0.9979	0.4~100	0.9938	0.5	1	/
239	Glipizide	+	21.04	446.2	321.2*/167	12/28	0.1~100	0.9963	0.1~100	0.9994	0.5	1	/
/	**Diuretics**	+											/
240	Bumetanide	+	21.96	365.1	240.1*/184	15/20	2~100	0.9947	2~100	0.9951	0.2	0.5	Chlorpromazine-D_6
241	Indapamide	+	18.5	366	132*/235	10/16	0.2~100	0.9993	0.2~100	0.9964	0.5	1	Chlorpromazine-D_6
242	Canrenone	+	22.1	341	107*/187	24/22	4~100	0.9912	4~100	0.9906	10	20	Chlorpromazine-D_6
243	Chlorthalidone	+	14.2	339	322*/241.2	10/25	10~100	0.9987	10~100	0.9908	2	5	Chlorpromazine-D_6
244	Triamterene	+	7.72	253.9	237*/104	23/30	1~100	0.9986	1~100	0.9940	0.8	2	/
245	Amiloride	+	4.57	230.1	171*/116.00	18/30	1~100	0.9912	1~100	0.9977	2	5	/

246	Cyclopenthiazide	+	24.53	380.1	205*/363.2	25/15	0.04~100	0.9981	0.04~100	0.9957	0.5	1	Chlorpromazine-D_6
/	**Pigment**												
247	Disperse-yellow 3	+	22.81	270.1	107.2*/150.2	25/15	0.04~100	0.9965	0.1~100	0.9884	0.2/0.5#	0.5/1.5#	/
248	Sudan 1	+	24.16	249.1	93.1*/232.2	12/20	0.4~100	0.9912	0.4~100	0.9913	0.5	1	/
249	Sudan 2	+	24.87	277.1	121.2*/156.1	20/15	0.04~100	0.9915	0.04~100	0.9934	0.5	1	/
250	Sudan blue 2	+	24.99	351.2	251.2*/294.2	30/18	0.04~100	0.9983	0.04~100	0.9971	0.2	0.5	/
251	Acid yellow 36	+	24.99	352.2	252.1*/295.2	30/20	0.04~100	0.9985	0.04~100	0.9981	0.2	0.5	/
252	Basic violet 1	+	21.99	358.2	342.2*/237.2	38/32	0.04~100	0.9912	0.04~100	0.9957	0.5	1	/
253	Crystal violet	+	22.31	372.2	356.2*/340.3	38/52	0.04~100	0.9912	0.04~100	0.9938	0.2	0.5	/
254	Rhodamine B	+	22.27	443.2	399.2*/355.1	42/58	0.04~100	0.9942	0.04~100	0.9945	0.2	0.5	/
/	**Pesticides**												
255	Fenthion sulfoxide	+	19.18	295	125.1*/201.2	30/15	1~100	0.9988	0.4~100	0.9921	4/2#	10/5#	/
256	Fipronil	-	5.61	435	250*/330	25/20	0.04~100	0.9968	0.04~100	0.9900	0.5	1	Fipronil-$^{13}C_2$，$^{15}N_2$
257	Fipronil-sulfone	-	6.37	451	282*/415	25/20	0.04~100	0.9911	0.04~100	0.9956	0.5	1	Fipronil-$^{13}C_2$，$^{15}N_2$
/	**Others**												
258	Dicyclomine	+	22.03	310.3	109.2*/165.2	25/18	0.04~100	0.9997	0.1~100	0.9901	0.2/0.5#	0.5/1#	/

259	4-Formylaminoantipyrine	+	6.45	232.1	214.2*/104.2	12/22	0.2~100	0.9987	0.2~100	0.9997	2	5	/
260	Aminophenzaone	+	5.13	232.1	113.2*/98	12/18	0.2~100	0.9976	0.04~100	0.9994	0.5/0.2#	1.5/0.5#	/
261	Antazoline	+	16.83	266.2	91*/196	25/15	0.04~100	0.9997	0.04~100	0.9996	0.2	0.5	/
262	Acetanilide	+	7.78	136.1	94*/77	15/25	0.04~100	0.9962	0.04~100	0.9981	0.5	1	/
263	Chlorpheniramine	+	14.75	275.1	230*/167	15/38	0.04~100	0.9983	0.04~100	0.9956	0.2	0.5	/
264	Doxepin	+	18.27	280.2	107*/235	22/18	0.1~100	0.9938	0.2~100	0.9966	0.5/1#	1/2#	/
265	Anastrozole	+	18.66	294.2	225.2*/142	20/38	0.04~100	0.9929	0.1~100	0.9992	0.2/0.5#	0.5/1#	/
266	Ketotifen	+	15.93	310.1	96*/82	20/32	0.04~100	0.9987	0.04~100	0.9996	0.2	0.5	/
267	Clomipramine	+	21.25	315.2	86*/58	15/28	0.04~100	0.9943	0.04~100	0.9969	0.2	0.5	/
268	Chloprothixene	+	21.14	316.1	271*/86	20/15	0.1~100	0.9984	0.1~100	0.9983	0.5	1	/
269	Citalopram	+	18.04	325.2	109*/262.2	22/18	0.04~100	0.9996	0.04~100	0.9966	0.5	1	/
270	Bromhexine	+	18.9	375	114*/216.8	18/28	0.4~100	0.9980	0.4~100	0.9932	0.2	0.5	/
271	Doxapram	+	15.17	379.2	292*/97	20/30	0.04~100	0.9993	0.04~100	0.9988	0.2	0.5	/
272	Dipyridamole	+	19.04	505.3	385.2*/429.2	42/42	0.4~100	0.9976	0.04~100	0.9921	4	10	/
273	Bromphenirami	+	15.52	319.1	274*/167	20/15	0.4~100	0.9981	0.4~100	0.9900	0.5	1	/
274	Zearalenone	-	4.19	317	175*/273	20/23	0.04~100	0.9972	0.04~100	0.9993	0.5	1	Zearalenol-D_4

275	Zearalenol	-	3.41	319	160*/275	30/20	0.04~100	0.9977	0.04~100	0.9900	0.5	1	Zearalenol-D_4
276	β-Zearalanol	-	3.34	321	277*/303	16/20	0.1~100	0.9966	0.04~100	0.9979	0.2/0.5#	0.5/1#	Zearalenol-D_4
277	α-Zearalanol	-	3.57			26/18	0.1~100	0.9971	0.04~100	0.9947	0.2/0.5#	0.5/1#	Zearalenol-D_4
278	Valnemulin	+	21.61	565.4	263.2*/164.2	16/30	0.1~100	0.9982	0.1~100	0.9965	0.5	1	/
279	Kresoxim-methyl	+	23.05	314.1	222*/167	8/15	4~100	0.9975	1~100	0.9923	10/5#	20/10#	/
280	N-Acetyl dapsone	+	12.07	291.2	108.2*/198.2	25/14	1~100	0.9942	1~100	0.9912	2	5	/
	**Internal standard**												
IS1	Dimetridazole-D_3	+	4.41	145	99.0*/83.0	16/20							
IS2	Metronidazole$^{15}N^{13}C$	+	3.92	176	132.0*/ 86.0	14/20							
IS3	Ronidazole-D_3	+	4.49	204	143.0*/58.0	10//22							
IS4	Clorprenaline-D_7	+	8.4	221	154.0*	22							
IS5	Cimaterol-D_7	+	5.39	227	161.0*	18							
IS6	Cimbuterol-D_9	+	5.39	244	162.0*	10							
IS7	Salbutamol-D_3	+	3.86	243	151.0*	16							
IS8	Sulfadiazine-D_4	+	4.63	255	160.0*	15							
IS9	Sulfamethoxazole-D_4	+	10.4	258	160.0*	14							

IS10	Clenbuterol-D_9	+	10.91	286	245.0*/204.0	15/15
IS11	Diazepam-D_5	+	21.92	290	154.0*/197.0	22/25
IS12	Chlorpromazine-D_6	+	20.94	325	92.0*/64.0	18/25
IS13	Sulfachlorpyridazine-$^{13}C_6$	+	9.12	291.1	162.0*/ 114.0	12/12
IS14	Ractopamine-D_5	+	8.91	307	121.0*/167.0	12/20
IS15	Enrofloxacin-D_5	+	9.87	365.1	321.0*/245.0	16/24
IS16	Sarafloxacin-D_8	+	12.2	394	303.0*/350.0	26/16
IS17	Tilmicosin-D_3	+	18.06	873	160.0*/696.5	40/40
IS18	Salmeterol-D_3	+	21.19	419.2	383.2.0*	16
IS19	Dienoestrol-D_6	-	4.12	271	95.0*	38
IS20	Diethylstilbestrol-D_8	-	4.12	275	259.0*/245.0	25/28
IS21	Estradiol-$^{13}C_2$	-	3.60	273	147.2.0*	43
IS22	Thiamphenicol-D_3	-	2.23	357	188.0*/293.0	12/18
IS23	Chloramphenicol-D_5	-	2.86	326	157.0*/262.0	11/17
IS24	Florfenicol-D_3	-	2.73	359	188.0*/339.0	10/18

IS25	Toltrazuril-D_3	-	5.37	427	427.0*	10
IS26	Lincomycin-D_3	+	6.35	410.2	362.*	18
IS27	Estrone-2,3,4-$^{13}C_3$	-	3.97	272	174.6*	37
IS28	17β-boldenone-D_3	+	0.92	290	121	22
IS29	Erythromycin-^{13}C-D_3	+	19.43	738.5	161.8*/580	30/18
IS30	Fipronil-$^{13}C_2$,$^{15}N_2$	-	6.37	439	251.8	28
IS31	Oxprenolol-D_7	+	15.05	273.2	79*/123	18/14
IS32	Atenolol-D_7	+	4.68	274	78*/144.7	16/16
IS33	Acebutolol-D_5	+	12.14	342.2	121*/222	18/15
IS34	Pindolol-D_7	+	6.43	256	123.1*/79.2	14/18
IS35	Nicarbazin-D_8	-	4.33	309.2	140.5*	11
IS36	Azithromycin-D_3	+	16.27	752.5	115*/594	38*/30
IS37	Megestrol-D_3 acetate	+	23.07	388.1	270.1*	18
IS38	Levonorgestrel-D_6	+	22.4	319.1	251.4*	30
IS39	Progesterone-D_9	+	23.16	324	100*	24

IS40	Methyltestosterone-D_3	+	23.19	306	109*	25
IS41	Testosterone-D_2	+	21.76	291	111*	20
IS42	17β-Boldenone-D_3	+	20.92	290	121*	22
IS43	Sulfadimethoxine-$^{13}C_6$	+	15.54	317	156*	20
IS44	Zearalenol-D_4	-	3.41	323	279	18

Note: "*" is the quantitative ion; "#" indicates the respective detection limits (LOD) or quantification limits (LOQ) of egg/muscle.

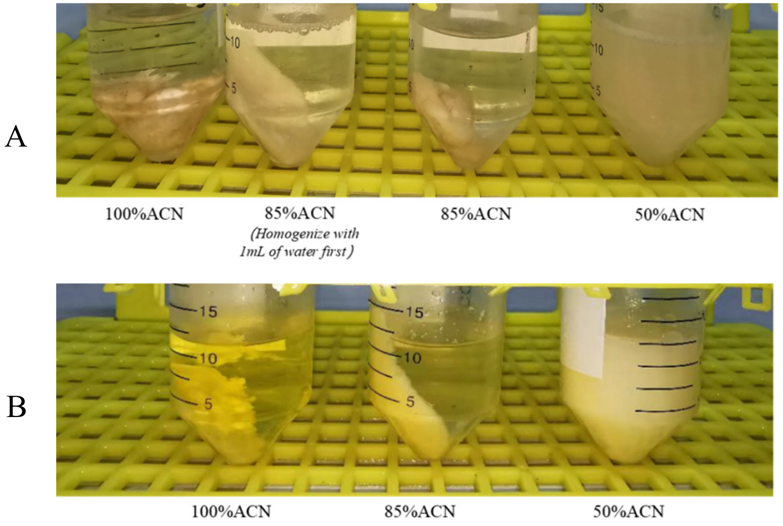

Figure S1 precipitation results using different acetonitrile-water solution for the extraction of chicken muscle (A) and eggs (B)
